# 3',4'-Dihydroxyflavonol has a positive effect on the cerebellum oxidant/antioxidant system and BDNF and transmitter levels in focal brain ischemia–reperfusion

**DOI:** 10.1007/s10863-026-10083-4

**Published:** 2026-05-01

**Authors:** Nisanur Coskun, Gozde Acar, Saltuk Bugra Baltaci, Ebru Kubra Uzdil, Tugce Aladag, Rasim Mogulkoc, Abdulkerim Kasım Baltaci

**Affiliations:** 1 Medical Faculty, Department of Physiology, Selçuk University, Konya, 42250 Türkiye; 2 Medical Faculty, Department of Physiology, Istanbul Medipol University, Istanbul, Türkiye

**Keywords:** Ischemia/Reperfusion, 3',4'-Dihydroxyflavonol treatment; MDA, GPx, Catalase, SOD, Thioredoxin, Glutamate, GABA and BDNF

## Abstract

Cerebral ischemia–reperfusion (I/R) occurs when blood flow is restored after a temporary interruption and may lead to brain dysfunction through oxidative stress and neurotransmitter imbalance. This study aimed to determine the effect of cerebral I/R and 1-week 3',4'-Dihydroxyflavonol (DiOHF) treatment on the antioxidant/antioxidant system and glutamate, GABA, and BDNF levels in cerebellum tissue in male rats. This study was performed on 28 Wistar-albino type male rats, which were formed as follows: 1-Control Group: No anesthesia or surgical procedure was applied to the animals in this group. 2-Sham Group: After general anesthesia was induced in the animals in this group, the carotid artery regions were opened and closed. After the application, solvent application was performed for 1 week (1 ml DiOHF vehicle). 3-Ischemia–Reperfusion Group: After general anesthesia, the carotid arteries were isolated in rats and ligated for 30 min, ischemia was induced, and then reperfusion was performed. 4-Ischemia–Reperfusion + DiOHF Group: After general anesthesia, the carotid arteries were ligated for 30 min and ischemia was performed in rats, then reperfusion was performed, and then DiOHF supplementation was performed for 1 week. After one week of treatment, the animals were sacrificed under general anaesthesia, and cerebellum tissues were taken. Malondialdehyde (MDA), glutathione peroxidase (GPx), catalase (CAT), superoxide dismutase (SOD), thioredoxin (TRx-2), glutamate, GABA, and brain-derived neurotrophic factor (BDNF) levels were evaluated in the relevant tissue. I/R increased MDA, glutamate, and GABA levels, while GPx, catalase, SOD, thioredoxin and BDNF levels were decreased. However, 1 week of 3',4'-Dihydroxyflavonol treatment corrected the changes that occurred with I/R. The study results show that 1 week of 3',4'-Dihydroxyflavonol treatment after I/R has a positive effect on cerebellum changes that occur in global brain I/R.

## Introductıon

Elucidating the pathophysiological mechanisms underlying stroke, which is a significant public health problem, with brain ischemia–reperfusion models and developing treatment strategies for these mechanisms are of great importance in terms of reducing neurological damage after stroke, supporting functional recovery, and improving long-term outcomes.

Brain ischemia is a significant and disabling neurological injury resulting from reduced cerebral blood flow (Salaudeen et al. 2024). Ischemia is a pathological process that occurs in neurological diseases such as traumatic brain injury and epileptic seizures (Lewerenz and Maher 2015). Ischemic stroke is mainly caused by blood clots that form in the cerebral artery and reduce cerebral blood flow to less than 10 mL/100 g/min, which in turn blocks blood flow to certain areas of the brain for several minutes (Blanco et al. 2022). Cerebral ischemia and reperfusion (I/R) is one of the most serious clinical manifestations of ischemic stroke and poses a significant burden on both individuals and society (Wang et al. 2022). Ischemic strokes constitute approximately 85% of all strokes and are mostly seen in middle-aged and elderly individuals, and are characterised by high incidence, mortality, and disability rates (Kaiser 2023).

The mechanisms that neutralize free radicals, prevent their formation, or scavenge existing free radicals from the environment are generally defined as the antioxidant system (Akkus et al. 1996). Although the activation of the antioxidant system generally increases at the beginning of ischemia, it is observed that this activity gradually decreases over time (Sadeghzadeh et al. 2023). Antioxidants administered early after the onset of ischemia can not only increase cell survival but also reduce the inflammatory response elicited by acute ischemia (Simion and Jurcau 2019). Oxidative stress plays a very important role in brain damage following cerebral I/R injury (Zhang et al. 2024). Oxidative stress is caused by reactive oxygen species (ROS) during cerebral ischemia and is more likely to lead to cell death and ultimately brain death after reperfusion. Especially during reperfusion, superoxide anion free radicals, hydroxyl free radicals, and nitric oxide (NO) are produced, which can cause lipid peroxidation, inflammation, and cell apoptosis (Wu et al. 2020). One of the main byproducts of lipid peroxidation is malondialdehyde (MDA) (Gaschler and Stockwell 2017).

Brain ischemia accelerates oxidative stress by decreasing endogenous antioxidants such as superoxide dismutase (SOD), catalase (CAT), and glutathione (GSH) and increasing malondialdehyde (MDA) levels (Ramakrishna et al. 2022). In a recent study, a significant increase in hippocampal MDA levels was detected in rats subjected to transient global cerebral ischemia/reperfusion. Treatment with the natural compound Cedrol reduced this increase in a dose-dependent manner and was found to be effective in suppressing oxidative stress (Asgharzade et al. 2025).

Some studies have shown that GPx provides more effective protection in antioxidative defence compared to SOD (Lee and Won 2014). In the study conducted by Heidari, Mahmoudzadeh-Sagheb, Sarbishegi and Gorgich (Heidari et al. 2021), a significant decrease in GPx, SOD, and catalase (CAT) activities was detected in the cerebellum; however, Withania coagulans extract (WCE) pretreatment significantly increased these enzyme activities and restored the oxidative balance.

Margaill, Plotkine and Lerouet (Margaill et al. 2005) reported a significant decrease in CAT activity at the beginning of reperfusion. While catalase activity was high in the hippocampus and lower cerebellum, GR and GPx activities were found to be high in the cerebellum and low in the hippocampus (Kumari Naga et al. 2007).

Thioredoxin (Trx), which has pleiotropic biological functions including cellular defence mechanisms against oxidative stress, is a small and ubiquitous protein (Lu and Holmgren 2014). Studies on cerebral ischemia have confirmed that increasing Trx2 levels can effectively reduce excessive oxidative stress and apoptosis, thereby alleviating the damage caused by cerebral ischemia–reperfusion (Lee et al. 2017). Superoxide dismutase (SOD) scavenges a large number of superoxide anion free radicals in the brain by surviving ischemia and reperfusion (I/R) damage, thus protecting cells and tissue from oxidative stress (Miao et al. 2017). There are findings that a significant decrease in SOD levels is observed in the weeks following ischemia (Asmah et al. 2022).

During the brain ischemia–reperfusion process, imbalances in the ratios of basic neurotransmitters such as glutamate and GABA directly affect the course of neuronal damage. A recent study reported that the Glu/GABA ratio increases significantly following ischemia, and this increase continues in different stages of reperfusion (Baranovicova et al. 2025). It is known that the cerebellum plays a strong role in the functional reorganisation of motor networks in stroke patients, especially in walking and balance functions (Koch et al. 2019). Although the cerebellum is classically considered a motor coordination centre, recent studies have highlighted its critical role in cognitive and affective processes. In fact, in patients with disorders confined to the cerebellum, significant alterations in neurological and mental functions have been clinically demonstrated, a condition described as the ‘cerebellar cognitive affective syndrome’ (Schmahmann and Sherman 1998). Therefore, cerebellar analysis in ischemia models may provide important insights into both motor and non-motor consequences of cerebral ischemia.” A previous study demonstrated that cerebral ischemia exerts a significant impact on the cerebellum by disrupting the migration and maturation of oligodendroglial progenitors, as well as the subsequent process of myelination (Barradas et al. 2016). In another study, cerebellar pericytes were shown to generate ischemia-induced multipotent stem cells that differentiate into active neurons and contribute to the regeneration of functional neuronal circuits (Beppu et al. 2019). The pathological mechanisms underlying cerebral I/R damage are complex and remain unclear (Shen et al. 2011). A series of events occurs in the brain tissue during ischemia or reperfusion, including cellular, biochemical, and metabolic consequences such as oxidative stress, overload of intracellular calcium ions, glutamate neurotoxicity, inflammation, and apoptosis (Bi et al. 2019). Therefore, assessing oxidative stress markers, neurotransmitters, and BDNF levels in the cerebellum provides valuable insights into ischemia–reperfusion injury beyond the forebrain and may help to explain functional outcomes observed after global ischemia.

BDNF was first identified in cerebral ischemia, promoting neurological function recovery and improving neurogenesis (Choi et al. 2015). Many studies have reported that BDNF levels are reduced after stroke (Casas et al. 2017). Flavonoids are an important class of secondary metabolites that are widely found in plants, contribute to plant growth and development, and have important applications in food and medicine (Liu et al. 2021). A growing body of literature reports the use of botanical medicine flavonoids in the prevention and treatment of cerebral ischemia reperfusion injury during ischemic stroke (Zhou et al. 2024). 3’,4’-Dihydroxyflavonol (DiOHF) is a synthetic flavonol that has been shown to not only exhibit antioxidant activity but also improve endothelial function in the aorta in the presence of oxidative stress (Chan et al. 2003). In this study, the effects of 3’,4’-Dihydroxyflavonol compound application for one week on the antioxidant defence system, BDNF level and neurotransmitter profile after ischemia–reperfusion injury in the cerebellum region were investigated. In the current literature, comprehensive studies on the neurobiological effects of this flavonoid, especially in the cerebellar region, are quite limited. In this respect, the study aims to shed light on the therapeutic potential of the relevant molecule and contribute to the development of new treatment approaches.

It has been demonstrated by previous studies that primary cerebral injury can induce secondary (remote) effects in the cerebellum in the absence of direct ischemic damage. Experimental models have shown that forebrain injury leads to apoptotic neuronal death in the cerebellum (Taylor et al. 2006; Parmar et al. 2024), while clinical studies have reported persistent or transient reductions in contralateral cerebellar blood flow following stroke, a phenomenon known as crossed cerebellar diaschisis (Liu et al. 2007). It is assumed that calcium influx into the cell mediated by canonical transient receptor potential (TRPC) ion channels plays an important role in the development of this secondary brain injury (Parmar et al. 2024).A recent study demonstrated that hypoxic–ischemic injury during the neonatal period is associated with long-term alterations in cerebellar activity that correlate with cognitive outcomes in adulthood (Sanches et al. 2019). Moreover, in studies examining microglial activation in the vermis and hemispheres of the cerebellum, marked astrogliosis was observed following hypoxic injury, while no significant differences in microglial phenotype were detected between postnatal day 3 (P3) and postnatal day 21 (P21) (Biran et al. 2011). These findings indicate that the cerebellum is responsive to hypoxic conditions and that certain glial responses persist across developmental stages.

Importantly, previous studies have shown that focal forebrain injury can induce apoptotic processes in anatomically remote brain regions, including the cerebellum, as a consequence of altered cerebral metabolism and disrupted neuronal connectivity (diaschisis) (Taylor et al. 2006). Consistent with this concept, in our earlier work, apoptotic activity in cerebellar tissue following ischemia–reperfusion was confirmed using hematoxylin–eosin staining, TUNEL labelling, and caspase-8 activity assays (Dasdelen et al. 2024). In a subsequent study, we further demonstrated that ischemia–reperfusion injury triggers endoplasmic reticulum stress in the cerebellum, as evidenced by altered expression of GRP78, HSP70, CHOP, Bcl-2, and Bax (Akkaya et al. 2025).

Taken together, these findings support the notion that the cerebellum, while not a primary ischemic target, undergoes subtle but biologically meaningful secondary alterations following cerebral ischemia–reperfusion. However, the effects of 3′,4′-dihydroxyflavonol (DiOHF) on the oxidant/antioxidant balance, BDNF levels, and neurotransmitter systems in cerebellar tissue have not yet been investigated. Therefore, the present study was designed to address this gap and to elucidate the potential protective and modulatory effects of DiOHF in the cerebellum under ischemia–reperfusion conditions.

## Materıals and method

This study was performed with the ethical approval of ethic committee of Selçuk University Experimental Medicine Application and Research Centre, dated 28.06.2024 and numbered 2024–42. In the study, 28 male Wistar-albino rats (Fakharaldeen et al. 2023; Jabbari et al. 2023), 10–12 weeks old and weighing an average of 300–400 g, obtained from the same centre, were used. Before the experiment, all animals were given a standard laboratory diet and free access to water. The rats were housed in a controlled environment with a temperature of 22 ± 2 °C and a 12-h light/12-h dark cycle. Biochemical analyses were conducted in the Molecular Physiology Laboratory of the Physiology Department of Selçuk University Faculty of Medicine.

### Creation of experimental groups

In the study conducted on a total of 28 Wistar-albino male rats, the groups were formed as follows.

1-Control Group (n = 6): No anaesthesia or surgical procedure was performed on the animals in this group.

2-Sham Group (n = 6): After general anaesthesia was induced in the animals in this group, the carotid artery regions were opened and closed. After the application, solvent application was performed for 1 week (1 ml DiOHF vehicle).

3-Ischemia–Reperfusion (I/R) Group (n = 8): After the carotid arteries were isolated in rats under general anaesthesia, they were ligated for 30 min, and ischemia was performed, followed by reperfusion.

4-Ischemia–Reperfusion + DiOHF Group (n = 8): After general anaesthesia, carotid arteries were ligated for 30 min, and ischemia was induced in rats, then reperfusion was performed, and DiOHF supplementation was performed for 1 week.

### Procedures performed on animals

Animals to be formed in Groups 2, 3, and 4 were treated with Ketamine HCl 60 mg/kg and xylazine HCl 5 mg/kg mixture intraperitoneally, and general anaesthesia was performed. Following this, the right and left carotid arteries were carefully isolated from the vagus nerve and surrounding tissues through a midline ventral neck incision in Group 2. Sham operation and vehicle supplementation (1 ml-hazelnut oil and 10% DMSO) (1 week) were performed in Group 2, and vehicle (1 ml-hazelnut oil and 10% DMSO) was applied in Group 3 (1 week) following I/R.

In Groups 3 and 4, the carotid arteries were ligated, and global transient cerebral ischemia lasting 30 min and then reperfusion was created. Following reperfusion, 10 mg/kg DiOHF (Fan Gaskin et al. 2022; Leo et al. 2011) was administered intraperitoneally for 1 week to the animals in Group 4. At the end of the experiments, animals were killed by cervical dislocation after taking blood from their hearts under general anaesthesia, and cerebellum tissues were taken from the brain. MDA, SOD, GPx, Catalase, TRX-2, Glutamate, GABA, and BDNF levels were evaluated in these samples.

### Biochemical analyses

#### Preparation of tissues

Tissue weights were recorded by weighing before homogenization. The tissue was cut into small pieces and placed in glass test tubes. It was homogenised in Misonix’s Microscan ultrasonic tissue disintegrator at 4 °C to form a 10% homogenate in PBS (pH: 7.4) (tissue weight (g): PBS (ml) volume = 1:9). The homogenates obtained were centrifuged at 5000xg for 5 min. The supernatants were transferred to clean tubes.

#### Determination of malondialdehyde (MDA) levels

It was carried out based on the method described by Mihara and Uchiyama (Mihara and Uchiyama 1978). Findings were evaluated in nmol/g tissue (Mihara and Uchiyama 1978).

#### Elisa analysis

To determine the levels of SOD (ng/g tissue), Cat (ng/g tissue), GPx (pg/g tissue), Glutamate (mcg/g tissue), GABA (nmol/g tissue), BDNF (ng/g tissue) and TRx-2 (ng/g tissue) in cerebellum tissues, rat SOD (E0168Ra, BT Lab, China), TRx-2 (E3511Ra, BT Lab, China), GPX (E1242Ra, BT Lab, China), CAT (E0869Ra, BT Lab, China), Glutamate (E1474Ra, BT Lab, China), GABA (E0102Ra, BT Lab, China), BDNF (E0476Ra, BT Lab, China) ELISA kits (BT Lab, China) and BMG Labtech device (Germany) as ELISA reader were used.

### Statistics

Statistical interpretation of the findings was performed using the SPSS 26.0 computer package program, and arithmetic means and standard deviations for all parameters were calculated. The Shapiro–Wilk test was performed to determine data homogeneity, and a normal distribution was determined. One-way analysis of variance (ANOVA) was used to determine the differences between groups, and the Duncan test, a multiple comparison test, was used to determine the origin of the differences. Differences at P < 0.05 were considered significant.

## Results

In our study, oxidant/antioxidant parameters in cerebellum tissue samples of rats and glutamate, GABA, and BDNF levels in the same tissue were determined.

**Fig. 1:**
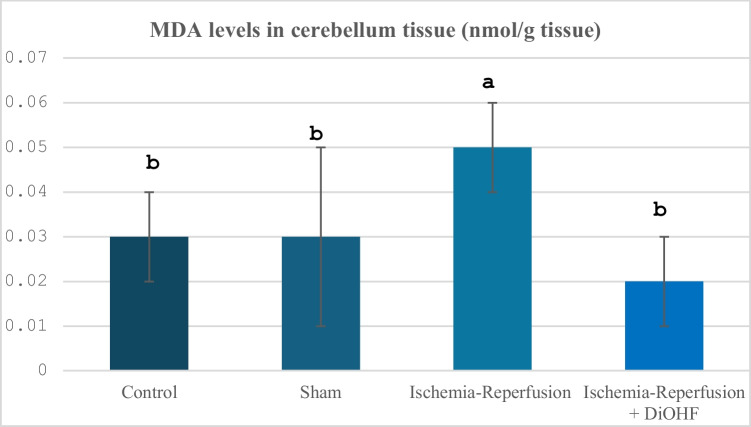
Distribution of MDA levels by spectrophotometric analysis according to groups. Different letters are significant as statistisc (a > b; P < 0.001). I/R increased MDA levels, however, DiOHF supplementation reduced

Table 1 and Fig. 1 show the MDA values ​​by group. When these values ​​were examined, it was seen that MDA levels increased after ischemia–reperfusion, while 1-week DiOHF treatment suppressed MDA levels (P < 0.001).

**Table 1:** Cerebellum MDA values in experimental groups

**Groups**	(Mean ± SD)
Control Group (n = 6)	0,03 ± 0,01 ^b^
Sham Group (n = 6)	0,03 ± 0,02^b^
Ischemia–Reperfusion (I/R) Group (n = 8)	0,05 ± 0,01 ^a^
Ischemia–Reperfusion + DiOHF Group (10 mg/kg) (n = 8)	0,02 ± 0,01 ^b^

The data are presented as mean ± standard deviation. A statistically significant difference exists between the groups identified by different letters (a > b; P < 0.001)

**Fig. 2:**
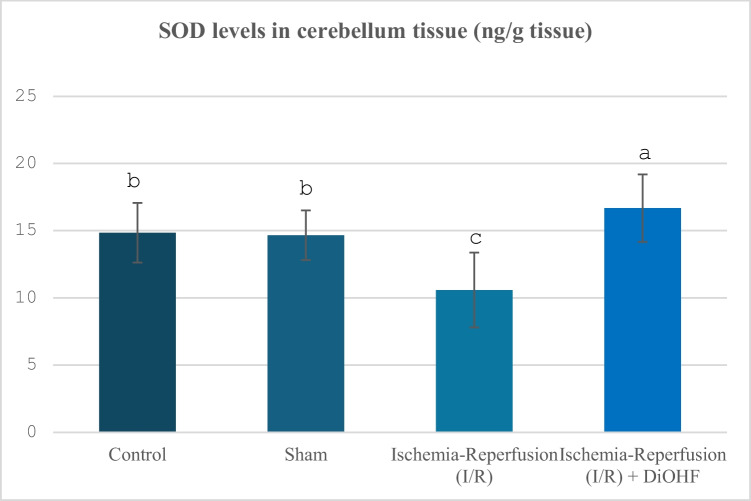
Distribution of SOD levels determined by ELISA analysis according to groups. Different letters are significant as statistisc (a > b > c; P < 0.001). I/R decreased SOD levels, but, DiOHF supplementation increased

SOD values ​​by group are shown in Table 2 and Fig. 2. While SOD levels decreased in the cerebellum after ischemia–reperfusion, 1-week DiOHF application provided a significant increase in these levels (Table 2; Fig. 2; P < 0.001).

**Table 2:** Cerebellum SOD values in experimental groups

**Groups**	(Mean ± SD)
Control Group (n = 6)	14,85 ± 2,22 ^b^
Sham Group (n = 6)	14,66 ± 1,85 ^b^
Ischemia–Reperfusion (I/R) Group (n = 8)	10,59 ± 2,78 ^c^
Ischemia–Reperfusion + DiOHF Group (10 mg/kg) (n = 8)	16,68 ± 2,5^a^

The data are presented as mean ± standard deviation. A statistically significant difference exists between the groups identified by different letters (a > b > c; P < 0.001)

**Fig. 3:**
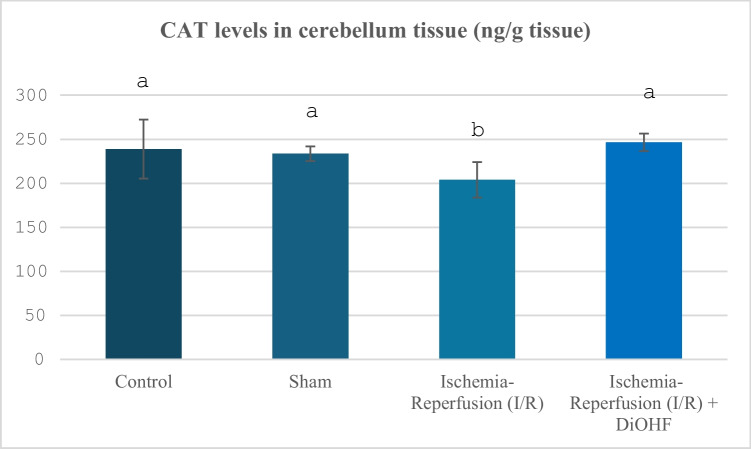
Distribution of CAT levels determined by ELISA analysis according to groups. Different letters are significant as statistisc (a > b; P < 0.001). I/R decreased CAT levels, but, DiOHF supplementation increased

Table 3 and Fig. 3 include the CAT values ​​measured by groups. It is seen that ischemia–reperfusion decreased CAT levels, and DiOHF application significantly improved this decrease (P < 0.01).

**Table 3:** Cerebellum CAT values in experimental groups

**Groups**	(Mean ± SD)
Control Group (n = 6)	238,8 ± 33,4 ^a^
Sham Group (n = 6)	233,54 ± 8,43 ^a^
Ischemia–Reperfusion (I/R) Group (n = 8)	203,96 ± 20,14 ^b^
Ischemia–Reperfusion + DiOHF Group (10 mg/kg) (n = 8)	246,39 ± 9,90 ^a^

The data are presented as mean ± standard deviation. A statistically significant difference exists between the groups identified by different letters (a > b; P < 0.01)

**Fig. 4:**
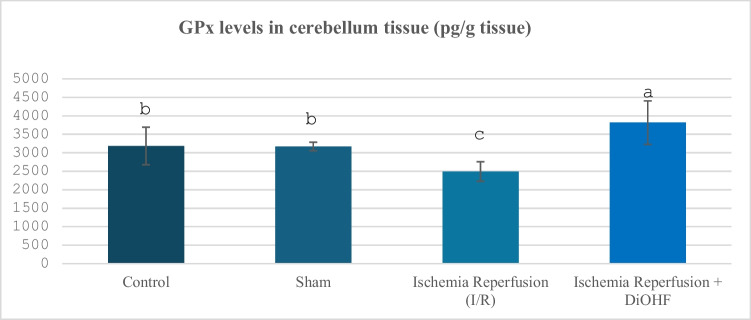
Distribution of GPx levels determined by Elisa analysis according to groups. Different letters are significant as statistisc (a > b > c P < 0.001). I/R decreased GPx levels, however, DiOHF supplementation increased

The GPx parameters of the examined groups are given in Table 4 and Fig. 4. When the values ​​were examined, a decrease in GPx levels was observed in the cerebellum region of the rat brain after ischemia–reperfusion, while 1-week DiOHF treatment reversed this effect on GPx levels (P < 0.005).

**Table 4:** Cerebellum GPx values in experimental groups

**Groups**	(Mean ± SD)
Control Group (n = 6)	3183 ± 508 ^b^
Sham Group (n = 6)	3170,6 ± 115,1 ^b^
Ischemia–Reperfusion (I/R) Group (n = 8)	2493,1 ± 264 ^c^
Ischemia–Reperfusion + DiOHF Grubu (10 mg/kg) (n = 8)	3815 ± 988 ^a^

The data are presented as mean ± standard deviation. A statistically significant difference exists between the groups identified by different letters (a > b > c; P < 0.005)

**Fig. 5:**
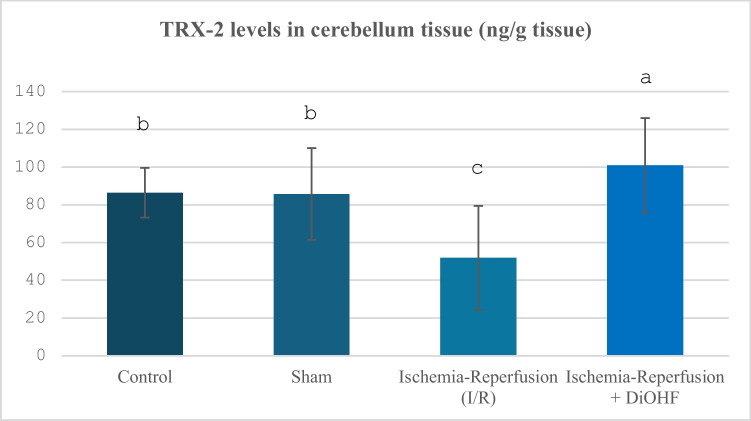
Distribution of TRx-2 levels determined by Elisa analysis according to groups. Different letters are significant as statistisc (a > b > c; P < 0.001). I/R decreased TRX-2 levels, but, DiOHF supplementation increased

Group data regarding the TRx-2 parameter are explained in Table 5 and Fig. 5. While I/R significantly suppressed cerebellum TRX2 levels, DiOHF supplementation increased them (Table 5; Fig. 5; P < 0.01).

**Table 5:** Cerebellum TRx-2 Values in Experimental Groups

**Groups**	(Mean ± SD)
Control Group (n = 6)	86,34 ± 13,17 ^b^
Sham Group (n = 6)	85,7 ± 24,3 ^b^
Ischemia–Reperfusion (IR) Group (n = 8)	51,8 ± 27,6 ^c^
Ischemia–Reperfusion + DiOHF Group (10 mg/kg) (n = 8)	100,9 ± 25 ^a^

The data are presented as mean ± standard deviation. A statistically significant difference exists between the groups identified by different letters (a > b > c; P < 0.01)

**Fig. 6:**
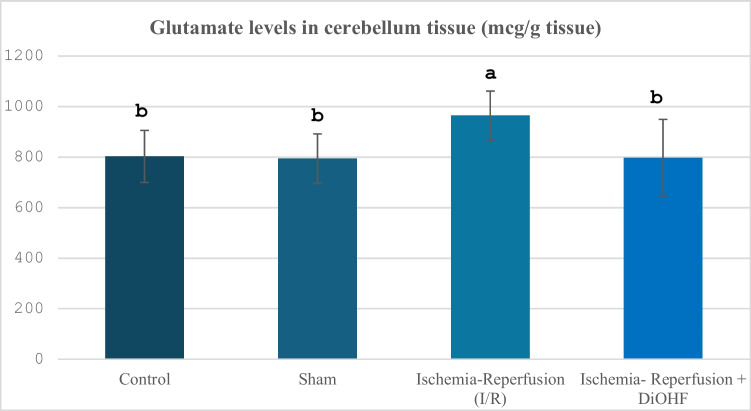
Distribution of Glutamate levels determined by Elisa analysis according to groups. Different letters are significant as statistisc (a > b > c; P < 0.001). I/R increased glutamate levels, but, DiOHF supplementation reduced

The glutamate values ​​according to the groups are shown in Table 6 and Fig. 6. While/R significantly increased cerebellum glutamate levels, DiOHF supplementation suppressed them (Table 6, Fig. 6).

**Table 6:** Cerebellum glutamate values in experimental groups

**Groups**	(Mean ± SD)
Control Group (n = 6)	802,6 ± 103,2^b^
Sham Group (n = 6)	794,5 ± 97,9 ^b^
Ischemia Reperfusion (IR) Group (n = 8)	964,2 ± 97,5 ^a^
Ischemia Reperfusion + DiOHF Group (10 mg/kg) (n = 8)	797,5 ± 151,4 ^b^

The data are presented as mean ± standard deviation. A statistically significant difference exists between the groups identified by different letters (a > b; P < 0.03)

**Fig. 7:**
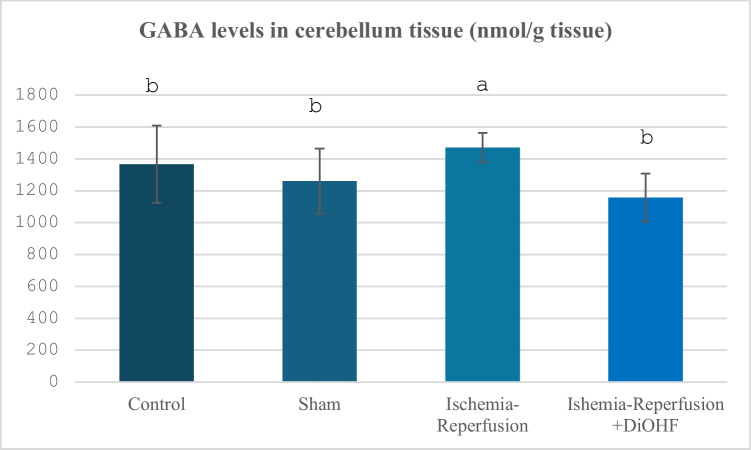
Distribution of GABA levels determined by Elisa analysis according to groups. Different letters are significant as statistisc (a > b >; P < 0.001). I/R increased glutamate levels, but, DiOHF supplementation reduced

The change in GABA values ​​according to the groups is shown in Table 7 and Fig. 7. Following ischemia–reperfusion, there was an increase in the amount of GABA in the cerebellum, and 1 week of DIOHF treatment corrected this situation (Table 7; Fig. 7; p < 0.01).

**Table 7:** Cerebellum GABA values in experimental groups

**Group**	(Mean ± SD)
Control Group (n = 6)	1365,6 ± 242,3 ^b^
Sham Group (n = 6)	1260,4 ± 203,7 ^b^
Ischemia/Reperfusion (IR) Group (n = 8)	1470,7 ± 92 ^a^
Ischemia–Reperfusion + DiOHF Group (10 mg/kg) (n = 8)	1156,7 ± 150,7^b^

The data are presented as mean ± standard deviation. A statistically significant difference exists between the groups identified by different letters (a > b; P < 0.01)

**Fig. 8:**
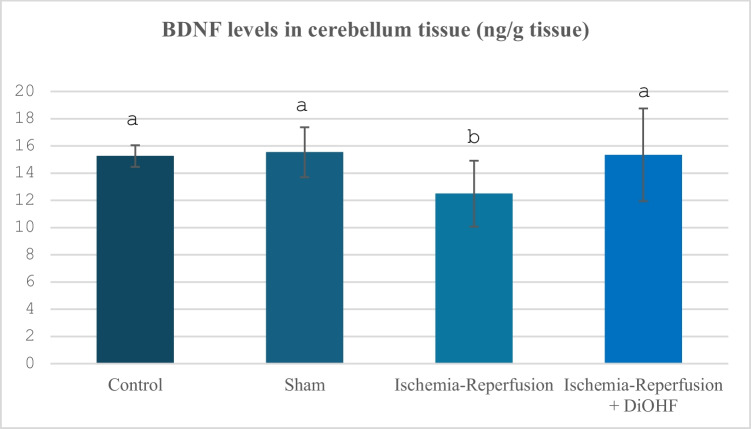
Distribution of BDNF levels determined by Elisa analysis according to groups. Different letters are significant as statistisc (a > b >; P < 0.001). I/R reduced BDNF levels, howver, one week DiOHF supplementation increased

Table 8 and Fig. 8 show the BDNF values ​​according to groups. When the values ​​were analysed, it was determined that BDNF levels decreased in the cerebellum region following ischemia–reperfusion, and DiOHF treatment increased these levels (P < 0.05).

**Table 8:** Cerebellum BDNF values in experimental groups

Groups	(Mean ± SD)
Control Group (n = 6)	15,26 ± 0,8 ^a^
Sham Group (n = 6)	15,54 ± 1,83 ^a^
Ischemia/Reperfusion (IR) Group (n = 8)	12,5 ± 2,42^b^
Ischemia/Reperfusion + DiOHF Group (10 mg/kg) (n = 8)	15,34 ± 3,40 ^a^

The data are presented as mean ± standard deviation. A statistically significant difference exists between the groups identified by different letters (a > b; P < 0.05)

## Dıscussıon

When the study was evaluated in general, as a result of global cerebral ischemia created by carotid artery occlusion in rats, oxidant system activity increased in the cerebellum tissue, while antioxidant system activity was suppressed. At the same time, neurotransmitter levels such as glutamate and GABA increased, but BDNF levels decreased. However, 1 week of DiOHF supplementation together with reperfusion after ischemia corrected the deteriorations that occurred with ischemia and reperfusion. In the study, firstly, changes in MDA levels in the cerebellum tissue were examined to determine the change in oxidant system activity, and while this parameter showed a significant increase due to ischemia–reperfusion, it was significantly suppressed with 1 week of DiOHF application afterwards. It is known that MDA levels increase after global cerebral ischemia–reperfusion. Oxidative stress is suppressed by decreasing MDA levels with the effect of various flavonoids. It is possible to find many studies in the literature in this direction. In one of these studies, Fang, Liu, Zhang and Song (Fang et al. 2024), it was reported that Baicalein, a type of flavonoid, significantly decreased MDA levels, a biochemical indicator of lipid peroxidation, in the cerebral ischemia–reperfusion model. It was reported that MDA values ​​​​were significantly reduced in animals treated with total oligomeric flavonoids prepared from C. Rotundus (Sunil et al. 2011). Jiang, Dai and Cui (Jiang et al. 2018) discovered that Vitexin reduced the expression of NO and pro-inflammatory cytokines as well as the level of lipid peroxidation marker MDA in rats with MCAO. Total flavonoid extract (TFDM) of Dracocephalum moldavica L. has a neuroprotective effect against cerebral ischemia–reperfusion injury. In this study, the effects of TFDM components on cerebral ischemia–reperfusion injury (CIRI) were evaluated both by network pharmacology and experimentally. TFDM treatment significantly reduced the level of MDA, one of the biochemical indicators of oxidative stress in brain tissue, and also increased NO activity and supported antioxidant defence (Hu et al. 2023). Guo, Mao, Li, Yu and Zhou (Guo et al. 2022) found that the combination of Ginkgo flavonoids and ginkgolides synergistically reduced the cerebral infarction rate and MDA content. It was reported that Ginkgolide B treatment significantly reduced the levels of malondialdehyde (MDA), an indicator of oxidative stress in brain tissue in rats with an ischemia/reperfusion model. In the analyses performed 24 h after reperfusion, a significant decrease in MDA levels was observed in the Ginggolide B-treated group (Cai et al. 2022). In a study conducted by Supawat, Palachai and Jittiwat (Supawat et al. 2025), it was determined that galangin, a flavonoid found in Alpinia officinarum, supplemented for 7 days against cerebral ischemia induced by the MCAO model, significantly reduced MDA levels. Silymarin was loaded into chitosan nanoparticles and applied as a pretreatment to evaluate its protective effects against cerebral ischemia/reperfusion injury. The treatment was given 14 days before ischemia and led to a significant decrease in MDA levels (Moghaddam et al. 2020). In our study, the suppression of the increase in lipid peroxidation product MDA levels resulting from ischemia–reperfusion with 1-week DiOHF application after ischemia–reperfusion is similar to the studies in question. Indeed, in similar experimental studies conducted previously, the suppression of MDA levels caused by even a single dose of DiOHF supplementation supports the current research findings (Dasdelen et al. 2021).

In our study, a large number of parameters were evaluated to determine the changes in antioxidant system activities in the cerebellum tissue, including SOD, GPx, Trx 2, and catalase. While SOD values ​​in the cerebellum tissue were suppressed by ischemia–reperfusion, DiOHF treatment caused an increase in the suppressed SOD levels. In a previous study conducted by Zhang, Yao, Qi, Song, Wang, Li, Zhou, Chang, Huang and Li (Zhang et al. 2023), it was reported that puerarin, which contains excess flavonoids, reduces oxidative stress by activating the PI3K/Akt/Nrf2 pathway and increases SOD activity. Calycosin increases SOD levels and suppresses TNF-α. In this way, it is seen that it is an effective flavonoid in reducing oxidative stress (Lu et al. 2022). In their study, Sunil, Kesavanarayanan, Kalaivani, Sathiya, Ranju, Priya, Pramila, Paul, Venkhatesh and Babu (Sunil et al. 2011) showed that superoxide dismutase (SOD) levels, one of the oxidative stress markers, decreased significantly after ischemia, but this decrease was significantly reversed in groups treated with total oligomeric flavonoids prepared from C. rotundus. It is observed that luteolin provides scavenging oxygen-free radicals in the mitochondria of rats exposed to middle cerebral artery occlusion (MCAO) and increases superoxide dismutase (SOD) activity through activation of the SIRT3/AMPK/mTOR signalling pathway (Liu et al. 2020). Application of Melastoma dodecandrum, a medicinal plant containing flavonoids, provided a significant increase in SOD levels in MCAO rats and contributed to the suppression of oxidative stress (Liu et al. 2025). Silymarin, whose protective effects against cerebral ischemia/reperfusion injury were evaluated, was loaded into chitosan nanoparticles and applied as a pretreatment. SOD levels increased with treatment given 14 days before ischemia (Moghaddam et al. 2020). Kaempferol treatment increased SOD levels after ischemia–reperfusion (Wang et al. 2020). It has been reported that SOD activity, which decreased due to I/R injury, increased significantly after apigenin treatment (Albrakati 2023). Icariin is a flavone molecule, and a significant increase in SOD levels was observed in the cortex of rats treated with icariin (Zahra et al. 2024). Similar to our study, SOD activity, which was suppressed in 2-h ischemia and 2-h reperfusion in ischemia–reperfusion (ovary), increased with DiOHF treatment. The increase in SOD activity, which was suppressed in the cerebellum tissue with ischemia–reperfusion, with DiOHF, a synthetic flavonoid, is similar to the effect of supporting SOD activation previously obtained with different flavonoids. Another antioxidant system indicator examined in the study, catalase levels, showed a significant decrease with brain-ischemia reperfusion. The current study is important in terms of revealing the negative effects of global cerebral ischemia on the cerebellum tissue in rats, and the antioxidant system activity showed suppression. GPx activity, one of the antioxidant activity indicators, was also examined in the study, and this parameter showed a significant suppression with ischemia–reperfusion. In a study, the decrease observed in glutathione peroxidase (GPx) levels after I/R was significantly improved with apigenin-supported treatment (Albrakati 2023). It has been reported that glutathione peroxidase (GPx) activity decreases after traumatic injury, but fisetin treatment significantly prevents this decrease (Zhang et al. 2018). It has been determined that 30-day E. ribes flavonoid extract application significantly increases GPx levels that decrease after ischemia (Nazam Ansari et al. 2008). Several studies suggest that drugs may help protect and maintain neurons by supporting or increasing antioxidant levels such as glutathione peroxidase (GPx), catalase (CAT), and superoxide dismutase (SOD), especially following Global cerebral ischemia (GCI) (Sadeghzadeh et al. 2023). Some studies have shown that GPx provides more effective protection in antioxidative defence compared to SOD (Lee and Won 2014). In a previous study, the decrease in catalase activity caused by cerebral ischemia–reperfusion was increased again by the administration of Melastoma dodecandrum Lour. Extract (Liu et al. 2025). In another study, silymarin loaded into chitosan nanoparticles administered prophylactically increased the activity of antioxidant enzymes such as CAT (Moghaddam et al. 2020). Treatment with apigenin causes significant increases in catalase (CAT) activities in rats (Albrakati 2023). It has been observed that rats treated with Icariin (ICA), a flavone molecule, caused a significant decrease in cerebral cortex MDA; on the other hand, a corresponding increase in cortex CAT levels was observed (Zahra et al. 2024). In our study, the increase in catalase activity was caused by cerebral ischemia–reperfusion.

In our present study, changes in thioredoxin levels were finally examined to evaluate antioxidant system activity, and this parameter showed significant suppression during ischemia–reperfusion, while DiOHF treatment prevented this suppression. It has been reported that increasing Trx2 expression can alleviate neuronal apoptosis, excessive oxidative stress, and cerebral ischemia–reperfusion injury. Thioredoxins (Trxs), the main antioxidants in the mitochondrial respiratory system, play important roles in reducing ROS by reversibly reducing Prx3 in the brain (Hwang et al. 2010). Intravenous administration of Trx has been reported to provide neuroprotection against ischemic stroke injury and improve survival (Mitsui et al. 2002; Hattori et al. 2004). Studies on cerebral ischemia have also confirmed that increasing Trx2 levels can effectively reduce excessive oxidative stress and apoptosis, thereby alleviating damage caused by cerebral ischemia–reperfusion (Lee et al. 2017). In a study conducted on transgenic mice expressing high levels of Trx, it was shown that Trx provides significant neuroprotective effects against ischemic brain damage (Takagi et al. 1999). These findings suggest that Trx plays a strong antioxidant role by suppressing the harmful effects of reactive oxygen species in the brain (Lee et al. 2017). Hwang, Yoo, Kim, Lee, Choi, Kwon, Kim, Choi and Won (Hwang et al. 2010) investigated the potential protective effect of Trx2, one of the mitochondria-specific antioxidant proteins, against ischemic damage in gerbils, in which they created a transient global cerebral ischemia model. In the study, it was shown that Trx2 levels were significantly decreased compared to the control group on the 1 st and 3rd days after ischemia, but Trx2 treatment administered intraventricularly suppressed this decrease and preserved the mitochondrial redox balance. Preservation of Trx2 levels after treatment reduced neuronal loss in the CA1 region and led to a significant decrease in apoptosis indicators (e.g., cytochrome c release, TUNEL-positive cells). These findings show that Trx2 is suppressed after ischemia, but oxidative stress can be reduced and neuronal death can be prevented with external supplementation. Sun, Hao, Li, Yin, Zong, Zhang and Gao (Sun et al. 2013) applied 15 min of ischemia followed by reperfusion for various periods in mice, focusing particularly on the hippocampal CA1 region. It was observed that Trx2 levels increased significantly after reperfusion in rats that received I/R. In a study evaluating the effects of tetramethyl pyrazine (TMP) treatment on the antioxidant defence system in the MCAO model created in Sprague–Dawley rats. And a significant decrease in Trx and TrxR gene expressions was detected after ischemia/reperfusion, but TMP treatment (20 mg/kg) reversed this decrease and strengthened the antioxidant defence (Jia et al. 2009). Flavonoids are natural antioxidants belonging to the polyphenol class. Resveratrol, a polyphenol, acts as a mitochondrial antioxidant by increasing the levels of the antioxidant trioredoxin-2 (TRx-2) and the X chromosome-linked apoptosis inhibitor protein (Kairisalo et al. 2011). Thioredoxin has a regulatory and antioxidant function in redox signalling. In a study, male Sprague Dawley rats (210–230 g) were injected intraperitoneally with vehicle or quercetin (10 mg/kg) (Fan Gaskin et al. 2022; Leo et al. 2011) 1 h before middle cerebral artery occlusion (MCAO). It was investigated whether quercetin regulates thioredoxin expression in global cerebral ischemia and glutamate-induced neuronal cell death. When the results were evaluated, thioredoxin expression decreased in vehicle-treated MCAO animals, while quercetin attenuated this decrease. Moreover, quercetin treatment attenuated the decrease in the number of thioredoxin-positive cells in the cerebral cortex of MCAO animals (Park et al. 2020). The fact that the decreased TRx 2 activity in the experimental global brain-ischemia reperfusion we performed was increased with DiOHF, whose effect has not been examined in any previous study, stands out as an important finding.

In our study, the effects of global cerebral ischemia–reperfusion on the levels of glutamate and GABA, which are important transmitters in the central nervous system, in the cerebellum tissue were also investigated, and both parameters showed significant increases as a result of global cerebral ischemia–reperfusion. In a previous study, flavonoids have been reported to have the potential to be clinically beneficial for retinal ganglion cell morbidities associated with hypoxia, glutamate, or oxidative stress (Nakayama et al. 2011). Sunil, Kesavanarayanan, Kalaivani, Sathiya, Ranju, Priya, Pramila, Paul, Venkhatesh and Babu (Sunil et al. 2011) reported that treatment with total oligomeric flavonoids prepared from C. rotundus significantly decreased glutamate levels in a rat model in which they applied 2-h MCAO followed by 70-h reperfusion, compared to the ischemic group. The suppression of glutamate neurotoxicity suggests that total oligomeric flavonoids contribute to neuronal protection by preventing excitotoxic damage. It is known that both naturally occurring and synthetic flavonoids have multiple effects on the activation of ionotropic receptors for GABA (Hinton et al. 2017). In their study, Islam, Hossain, Ahmed, Rahaman, Al-Khafaji, Khan, Sarkar, Bappi, de Andrade, Araújo, Coutinho, Kowalska, Kowalski, Hanif and Islam (Islam et al. 2022) showed that quercetin, a flavonoid compound, interacts with GABAA receptor subunits and produces anxiolytic-like effects, and this effect is associated with high binding affinity to GABA α5, β1, and β2 receptor subunits. This finding suggests that flavonoids, known to have a regulatory effect on the GABA system in ischemic conditions, may be potential therapeutic agents. A study has shown that a flavonoid compound called malvidin has a neuroprotective effect by targeting the GABA signalling pathway in ethanol-induced memory disorders. In this study, the interaction of Malvidin with GABA receptors was confirmed by molecular docking analyses, and it was shown that learning and memory functions were improved through modulation of the GABAergic system (Bawadood et al. 2024). In the experimental findings of the present study, the initial increase in neurotransmitter activity is similar to some previously conducted studies, while in some studies, there are different findings (decrease) especially for GABA, but when the studies in the literature are examined, it is determined that the changes in GABA levels differ according to the ischemia–reperfusion period and the analysis time afterwards. In our study, the increased glutamate and GABA levels due to ischemia–reperfusion were suppressed with 1 week of DiOHF treatment, which is important in terms of suppressing glutamate toxicity. Finally, in our study, the changes in BDNF levels in cerebral global ischemia–reperfusion were examined, and this parameter showed significant suppression with I/R. However, the treatment brought the suppressed BDNF levels back to normal levels. Improvements in human cognitive function provided by dietary flavonoids may be mediated, in part, by increased BDNF expression in both the brain and the periphery, and serum levels of this neurotrophin may also act as a potential biomarker of cognitive performance in humans (Neshatdoust et al. 2016). The neuroprotective effects of heptamethoxyflavone (HMF), a citrus-derived flavonoid, were investigated in mice with a cerebral global ischemia model. In this study, it was reported that HMF treatment significantly increased brain-derived neurotrophic factor (BDNF) levels, especially in the hippocampus after ischemic damage. In addition, it was stated that this increase in BDNF expression contributed to the recovery of cognitive functions such as learning and memory following the ischemic process. These findings support that the regulation of BDNF via flavonoids may be a potential therapeutic approach against cerebral ischemia (Okuyama et al. 2012). In a study conducted on rats using MCAO, a significant decrease was observed in the number of BDNF and tropomyosin receptor kinase B (TrkB)- positive cells, especially in the ischemic cortex, as of the 7th day. It has been reported that quercetin treatment significantly increased the number of BDNF and TrkB positive cells in the ischemic cortex and supported neuronal survival (Yao et al. 2012). Previous studies have determined that different flavonoids affect BDNF activity during global cerebral ischemia–reperfusion via various kinases. Indeed, subcutaneous administration of 3,5,6,7,8,3',4'-heptamethoxyflavone (HMF), a citrus polymethoxyflavone, orally administered HMF is beneficial for the upregulation of BDNF in the hippocampus via the extracellular signal-regulated kinase 1/2 (ERK1/2)/MAP system (Sawamoto et al. 2017). In another experimental study, intragastric kaempherol administration at a dose of 25 mg/kg affected BDNF levels via the TrkB-PI3K/AKT signalling pathways (Zhang et al. 2024). In another study, administration of naringin at two different doses (50 and 100 mg/kg) affected CREB/BDNF signalling during global cerebral ischemia–reperfusion (Alijani et al. 2025). Although the activity of kinases related to these signalling pathways was not examined in our study, it is possible that they provided an increasing effect on BDNF levels through different kinase activities identified in previous studies. The fact that no previous study examining the relationship between 3',4'-dihydroxyflavonol and BDNF has been encountered highlights the originality of this study in this respect.

## Conclusıon and recommendatıons

When the results of the current study are examined, firstly, it is seen that MDA levels increase significantly in terms of oxidant system activity in global cerebral ischemia–reperfusion, while antioxidant system indicators such as SOD, catalase, GPx, and Thioredoxin levels are suppressed. At the same time, it is seen that there are increases in glutamate and GABA levels due to I/R. Also, BDNF levels, which are important for cognitive functions as a result of I/R, are suppressed significantly. However, 10 mg/kg DiOHF supplementation for one week after I/R corrected the negativities that occur due to global cerebral I/R in rats to a certain extent.

In this study, we determined that DiOHF supplementation after brain ischaemia–reperfusion inhibited MDA levels and increased the antioxidant activity of cerebellum tissue. And also regulate some transmitters such as glutamate and GABA. Meantime, DiOHF affects BDNF levels in this experimental model. These can be considered as concrete findings showing the potential therapeutic effects of DiOHF. All these suggest that DiOHF may be a powerful flavonoid, especially in protecting BBB integrity. However, this study includes some limitations and more detailed studies are needed in the future to elucidate the molecular mechanism in the related pathways. The activity of kinases related to these signalling pathways was not examined in our study; it is possible that it provided an increasing effect on BDNF levels through different kinase activities identified in previous studies. In particular, potential molecular pathways such as Nrf2, PI3K/Akt, and CREB, which may explain how DiOHF might exert these effects, have not been investigated. In the future, it will be possible to elaborate on the mechanisms by using different experimental models and more application routes/durations.

## Limitations

Histological evaluations could not be performed due to technical and logistical constraints, which should be considered a limitation of the study. In this study, molecular analyses were conducted without blinding; the fact that the investigator performing the analyses was not blinded to group allocation should be considered a methodological limitation of the study.

## Data Availability

Yes. The datasets generated during the current study are available from the corresponding author upon reasonable request.
